# COVID-19 Transition: Does virtual exercise maintain physical function?

**DOI:** 10.1093/geroni/igaa057.3440

**Published:** 2020-12-16

**Authors:** Stephen Jennings, Kenneth Manning, Megan Pearson, Catalin Mateas, Katherine Hall, Janet Bettger, Miriam Morey

**Affiliations:** 1 Durham VA Geriatric Research, Education, and Clinical Center (GRECC), Veterans Affairs Health Care System, Durham, North Carolina, United States; 2 Durham VA Health Care System, Durham, North Carolina, United States; 3 Veteran Affairs Health Care System, Durham, North Carolina, United States; 4 Duke University, Veterans Health Administration, Durham, North Carolina, United States; 5 Duke University School of Medicine, Durham, North Carolina, United States; 6 Veterans Health Administration, Durham, North Carolina, United States

## Abstract

Background: In March 2020, COVID-19 mandates to restrict face to face exercise and group based gatherings were enacted. These mandates were enforced within most states in the US. Gerofit, a facility-based exercise program for older Veterans in Durham, NC, transitioned to remote virtual exercise instruction to accommodate continuity of care. Objectives: To explore whether remote virtual exercise (RVE) can sustain physical function within individuals previously participating in onsite face to face exercise (OFF). Methods: Physical function assessments performed during OFF were compared with assessments conducted remotely over virtual platform. Assessments included the 30-second arm curl, the 30-second chair stand, time to complete five chair stands, and either 6-minute walk or 2-minute step test. All assessments for RVE were completed via a remote virtual platform. Only participants enrolled in both OFF and home based RVE with functional assessments within 6-months of pre and post COVID-19 transition were compared. Descriptive comparisons, opposed to statistical, were reported due to the limited sample size. Results: Fourteen OFF Gerofit participants were reassessed remotely within the first 6-months of transitioning to RVE (12 male, 2 female, mean age 73.1, mean body mass index 31.5). Functional assessments between OFF versus RVE were arm curls (21.0 vs 20.4 repetitions), chair stands (15.0 vs 17.5 repetitions), and time to 5 chair stands (9.0 vs 8.4 seconds). Cardiovascular function, reported in normalized percentiles (46.4%tile vs 58.9%tile) Conclusion: Among older Veterans engaged in regular structured exercise, physical function was preserved with transition to virtual exercise.

